# Immunomodulatory and anti-inflammatory effects of probiotics in multiple sclerosis: a systematic review

**DOI:** 10.1186/s12974-019-1611-4

**Published:** 2019-11-21

**Authors:** Mohammad Morshedi, Reza Hashemi, Sara Moazzen, Amirhossein Sahebkar, Elaheh-Sadat Hosseinifard

**Affiliations:** 1 Drug Applied Research Center, Tabriz University of Medical Sciences, Tabriz, Iran; 2 Department of Epidemiology, University Medical Center Groningen, University of Groningen, Groningen, 9713 GZ the Netherlands; 3 Neurogenic Inflammation Research Center, Mashhad University of Medical Sciences, Mashhad, Iran; 4 Biotechnology Research Center, Pharmaceutical Technology Institute, Mashhad University of Medical Sciences, Mashhad, Iran; 5 School of Pharmacy, Mashhad University of Medical Sciences, Mashhad, Iran

**Keywords:** Probiotics, Autoimmune diseases, Microbiome, Gut-brain axis, Gut microbiota, Multiple sclerosis, Immune/inflammatory response, Systematic review

## Abstract

Multiple sclerosis (MS) is an inflammatory and autoimmune neurological disorder which leads to demyelination. Although the etiology of MS is yet to be known, it appears that regulating the immune system and suppressing inflammatory pathways may possibly have a favorable effect on the healing of this disease. Evidence suggests that probiotics consumption via gut microbiome alteration devises beneficial effects in improving immune and inflammatory responses in MS. All articles were systematically searched (in the main databases) for this paper. Two investigators independently scrutinized full texts of the potentially eligible articles. The quality of the study was evaluated using standardized tools. The methodological quality of seven studies included in this review ranged from fair to good. The findings illustrated that there were statistically significant improvements in the static and dynamic balance in patients and animals with MS. In the paper in hand, the effects of probiotics administration on immune and inflammatory markers in MS disease are evaluated. In addition, the limitations and knowledge gaps were reported while proposing a possible mechanism of probiotics therapy in modulating immune and inflammatory responses. This systematic review indicated that the probiotics could improve immune and inflammatory parameters, the cytokines and cells in MS disease. Probiotics may have efficient effects in management and treatment of MS. More studies are required to clarify the effect of supplementation with probiotics and their mechanisms in MS disease.

## Background

Multiple sclerosis (MS) is considered to be a distinguished inflammatory, demyelinating and autoimmune neurological disorder affecting 2.5 million people worldwide [1]. Frequently observed symptoms are numbness, fatigue, vision loss, dizziness, cognitive defects, depression, and bowel dysfunction [1–4]. MS risk factors can be categorized into two types: genetic and environmental [1]. The key factors initiating MS, for the most part, continue to remain unknown [1, 5, 6]. MS pathology is characterized by an increase in inflammatory responses, destruction of myelin sheaths, proliferation of astrocytes, microglia activation, gliosis, and axonal degeneration [7, 8]. Additionally, numerous studies have confirmed that MS is an immune-mediated and inflammatory disease in which the myelin of the nerve cells is attacked by the immune system and mediated by the cluster of differentiation 4 (CD4) myelin-reactive T-helper 1 (Th1) cells [9–14]. Evidence has suggested that inflammation leads to degeneration of brain axons and neurons in people with MS. Anti-inflammatory and immunomodulatory treatments can prevent or delay the progression of the MS [11, 14–17]. Findings reveal that the gut microbiome may well have a notable role in the immune system regulation and exerting anti-inflammatory, antioxidant, and metabolic effects in the host [14, 18–21]. The latter suggests that targeting gut microbiome might be a crucial target for prevention, management, and control of the inflammatory and autoimmune diseases [22–25].

Evidences are accumulating to suggest that there is a bidirectional relationship between the intestine and the central nervous system (CNS), which is called the gut-brain axis [26]. Moreover, studies reported that alteration of the gut microbiome could influence inflammatory responses in human beings and animals with MS [23, 27, 28]. In this regard, Hoogen et al. [29] indicated that there was an interesting role of diet and gut microbiome in the modulation of immune diseases. Furthermore, this study determined whether MS could be treated via modification of gut microbiota and probiotics administration. On the other hand, Probiotics could have favorable effects on the host as functional food and good microorganisms [18] through normalization of the imbalance gut microbiome [30–32]. In addition, numerous studies have publicized that the use of probiotics could improve immune/inflammatory processes in some diseases such as type 2 diabetes [21], inflammatory bowel disease (IBD) [33], and neuroinflammatory disorders [34, 35]. In addition, some studies have exposed that probiotics intake could perform an efficient role in MS disease [35, 36]. *Lactobacilli* and *Bifidobacteria* are of the most common probiotics, which possess substantial health-promoting properties such as modulating the population and composition of gut microbiome and improving intestinal barrier function [21, 37, 38]. Furthermore, these microorganisms facilitate the production of metabolic parameters such as short-chain fatty acids (SCFAs) [39, 40] and reduce gut permeability [41], which ultimately leads to improved immune responses and decreased inflammation [21, 42, 43]. Effects of probiotics on MS are more commonly assessed in animal studies. Recently, a pilot study demonstrated that probiotics (*VSL*3) administration was associated with host immune system. Alteration of gut microbial composition by *VSL*3 supplementation had beneficial effects on the immune and inflammatory responses in patients with MS [44]. The mechanisms of the effects of probiotics administration on various features of MS are still largely undefined and require further investigation.

In this systematic review, the attempts were to evaluate the effects of probiotics consumption on human and animal models of MS and explore the mechanisms of effectiveness with a particular focus on the immune function and inflammation.

## Methods

The study was performed according to the recommendations of the Preferred Reporting Items for Systematic Reviews and Meta-Analyses (PRISMA) statement [45]. The systematic review was registered in PROSPERO, an international prospective register of systematic reviews (registration number, CRD42018086594).

### Search methods for identification of studies

PubMed, the Cochrane Central Register of Controlled Trials (CENTRAL), Scopus, EMBASE, Web of Science, Google Scholar, and reference lists of retrieved articles (prior to December 24, 2018) were systematically searched for the various combinations of the following terms: (Multiple Sclerosis or MS or Experimental Autoimmune Encephalomyelitis (EAE) or EAE or Auto-Immune Disease or Autoimmune Disorder) and (Lactobacillus or Lactobacillales or Probiotics or Lactobacilli or Lactic Acid Bacteria or Probiotic or Bifidobacteria or Bifidobacterium). The Cochrane Library and databases (included in this review) were searched to ensure there were no other systematic reviews on this topic.

### Inclusion criteria

Inclusion criteria: (1) all article written in English language; (2) interventional studies including RCTs and experimental studies, which assessed the effects of probiotics administration on immune or/and inflammatory markers in MS patients or animal models of MS (EAE); (3) studies on individuals diagnosed with MS; (4) studies reporting the association between probiotic intake and immune and inflammatory response. Also, the full text of potentially eligible articles was scrutinized independently by two investigators.

### Outcome measures

Safety and tolerability outcomes included the proportion of patients who experienced any treatment-emergent adverse events (AEs), those withdrawn from the treatment for AEs, and those experienced any of the following: agitation, anxiety, cutaneous rash, diarrhea, dysgeusia, edema, euphoria, fatigue, gastric pyrosis, headache, hiccup, hot flashes, hypertension, insomnia, nausea, palpitations, and vomiting. Available data on pharmacological bioavailability were extracted.

### Data extraction and quality assessment

The trials and animal studies were independently assessed by three authors for inclusion and the gained information from included trials were extracted by them as well, and, in the case of discrepancies, the final decision was made by a third investigator or by consensus. Studies were assessed based on the Population, Intervention, Comparators, Outcomes, Timing/Setting (PICOTS) framework [46]. Relevant outcomes including sample characteristics (region, population, age, gender), intervention characteristics (probiotic species and strains, duration of intervention, dose), and specific results (all immune and inflammatory markers) were extracted from the selected articles. The study quality and the risk of bias were critically evaluated using a standardized tool, which was the Cochrane Risk of Bias Tool to Agency for Healthcare Research and Quality (AHRQ) Standards and SYRCLE’s tool was used for assessing risk of bias for animal studies (Additional file 1) [47–49]. From each eligible article, the first author, journal, year of publication, reported risk factors, and number of studies were recorded. Any disagreement was resolved by consensus.

## Results

### Study selection

**Fig. 1:**
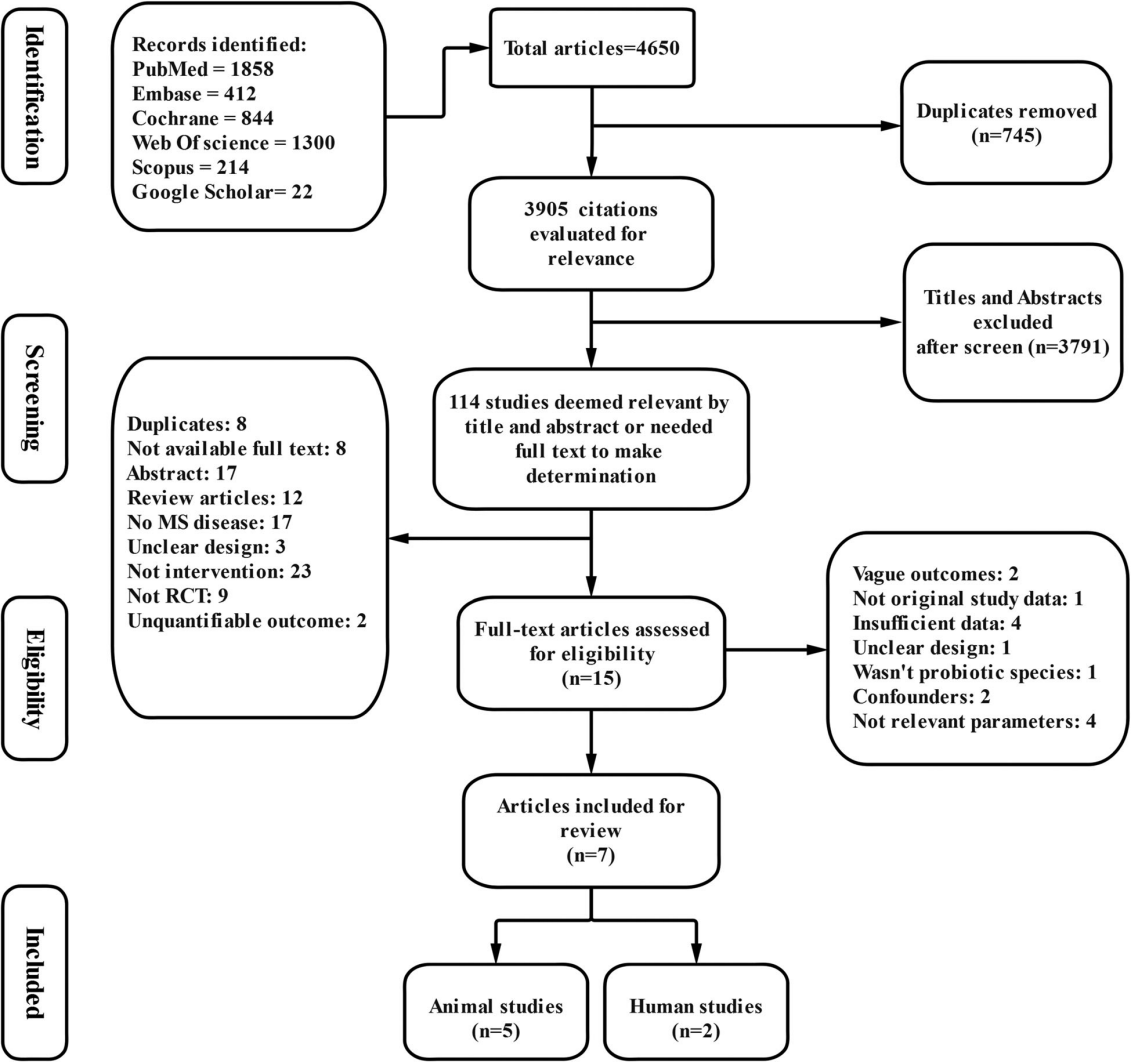
Collection of studies for inclusion in systematic review

A total of 4650 articles were initially retrieved as shown in Fig. 1. Three thousand nine hundred five citations remained after duplicate studies were excluded (745). According to mentioned reasons, 745 articles were deemed ineligible and were subsequently removed. Then, the titles and abstracts of the remaining articles were reviewed and 114 studies were deemed to be potentially eligible (3791 articles were excluded). After reviewing the full texts of the 15 candidate articles, seven eligible articles (2 RCTs and 5 animal studies) were finally included for the present systematic review.

### Study characteristics

Two RCTs and five animal studies were published in English language journals during February 2010 to January 2019. Three studies were carried out in Iran [35, 50], one in France [51], one in Japan [52], one in the Republic of Korea [53], and another in Sweden [54]. A summary of the study features and major results are depicted in Table 1. Both human studies were randomized, double-blinded, and placebo-controlled trial. Participants were all relapsing–remitting MS (RRMS) and identified according to McDonald criteria and an expanded disability status scale (EDSS) score ≤ 4.5, in the range age of 18–60 years with a majority of female participants, by 75–83%. The total number of individuals was in the range of 48–60. Whilst in animal studies, the female and male Myelin Oligodendrocyte Glycoprotein (MOG)-induced EAE in C57BL/6, C57/BL6J, and PLP (Proteolipid protein)-induced EAE in SJL/J mice were applied in the range age and number of 7–12 weeks and 20–70 odds, respectively. The intervention obtained probiotics including *L*. *plantarum* (*n* = 2) [35, 54], *B*. *animalis* (*n* = 1) [35], *E*. *coli* Nissle (*n* = 1) and archetypal (*n* = 1) [51], *L*. *casei* (*n* = 3) [50, 52], IRT5 (*n* = 1) [53], *L*. *paracasei*, *L*. *delbrueckii*, *L*. *bulgaricus* (*n* = 1) [54], *L*. *acidophilus*, *B*. *bifidum*, *L*. *fermentum* (*n* = 1) [50], and *B*. *infantis*, *B*. *lactis*, *L*. *reuteri* (*n* = 1) which was prepared in probiotic capsules for human studies and also, oral gavage for animal studies. The used dosage was 0.6–5 × 10^8^–10^9^ colony-forming units (CFU) for 22 to 50 days.

**Table 1:** Published research examining the effects of probiotics intake on immune and inflammatory markers in animal and human models of MS

Author	Sample	Supplementation	Duration	Measurements	Findings
Salehipour et al., 2017 [35]	MOG-induced EAE in Female C57BL/6 mice, 8–10 weeks old, *n* = 8 per group	Treatment (T) groups: T1: *L*. *plantarum* T2: *B*. *animalis* T3: Both of probiotics. 10^9^ CFU	22 days	- CD4+, CD25+, Foxp3+, IL-10, IL-4, TGF-β, GATA3 - IFN-γ, IL-17, IL-6, T-bet, and ROR-γt	T1, T2, and T3 (particularly in T3): - Mitigation of EAE in mice through motivating polarization of CD4+ T-cells toward T-reg by induction of anti-inflammatory cytokines and transcription factors and inhibition of pro-inflammatory cytokines, thereby suppressing autoreactive T-cells proliferation and leukocyte infiltration into CNS
Secher et al., 2017 [51]	MOG-induced EAE in male C57/BL6Jmice, 8–12 weeks old, *n* = 30–40 per group	T groups: T1: *E*. *coli Nissle* T2: archetypal. 10^8^ CFU	30 days	- Z0–1, Claudin-8, Reg3γ, Reg3β - CD4+, IFN-γ, IL-17, and TNF-α	- Reduced the severity of EAE induced by immunization with the MOG35-55 peptides - Decreased secretion of inflammatory cytokines and an increased production of the anti-inflammatory cytokine by autoreactive CD4 T-cells, in peripheral lymph nodes and CNS - Increased numbers of MOG-specific CD4+ T-cells in the periphery contrasting with severely reduced numbers in the CNS - Repaired intestinal permeability dysfunction
Kobayashi et al., 2012 [52]	PLP-induced EAE in SJL/J and MOG-induced EAE in C57BL/6 Female mice, 7 weeks old, *n* = 15 per group	- T group: *L*. *casei* 0.6–1.2 × 10^9^ CFU	50 days	- IL-17 and IFN-γ, IL-10 protein levels and mRNA, - CD4+, CD25+, CD8+ T-reg cells	- Non-significant neurological symptoms or histopathological changes of the spinal cord (SC) in either model - Improve neurological symptoms in the PLP-induced EAE in SJL/J mouse Unregulated IL-17 production by antigen-stimulated lymphocytes of draining lymph nodes on day 7 - Enhanced production of IL-10 and an increase in the percentage of CD4+CD25+ T-reg - Strong expression of IL-17 mRNA on day 12, but this expression was not enhanced by *LcS* administration
Kwon et al., 2013 [53]	MOG-induced EAE in C57BL/6mice 6–8 weeks old, *n* = 10 per group	The IRT 5 probiotics contains 10^8^ CFU/g of each strain: *L*. *casei*, *L*. *acidophilus*, *L*. *reuteni*, *B*. *bifidum*, *Streptococcus thermophiles*. (final 5 × 10^8^ CFU)	30 days	IL-4 (Th2) and IL-10 (Th2) and CD4+ T-cells, Foxp3+, IL-2 - CD3+, Gr1^+^ or/and CD11b^+^ monocyte, B220^+^ B cells - Th1/Th17; IFN-γ, TNFα and IL-17 protein levels and mRNA, IL-1β	- Prophylactic and therapeutic effects - A T-cell-mediated inflammatory autoimmune disease of the CNS - Pretreatment of IRT5 probiotics expressively suppressed EAE development - Delayed the disease onset of EAE - Inhibited the pro-inflammatory Th1/Th17 polarization, while inducing IL-10+ producing or/and Foxp3+ T-reg, both in the peripheral immune system and at the site of inflammation
Lavasani et al., 2010 [54]	MOG-induced EAE in Female C57BL/6mice, 8–10 weeks, *n* = 5 per group	T1: *L*. *paracasei*, DSM 13434; T2: *L*. *plantarum*, DSM 15312; T3: *L*. *plantarum*, DSM 15313; T4: *L*. *paracasei*, PCC, 101; T5: *L*. *delbrueckii*, subsp. *L*. *bulgaricus*, DSM 20081. T6: T1, T2, and T3. 10^9^ CFU	25 days	- Th2 type cytokines, - IL-4, IL-10, TGF-β1 CD25, CD4+T-cells IL-27, TNF-α, IFN-γ, IL-17	- T1, T2, and T3 reduced inflammation in CNS and autoreactive T-cell responses - T1 and T2 induced CD4+CD25+Foxp3+ T-reg in MLNs and enhanced production of serum TGF- β1, while T3 increased serum IL-27 levels - T6 suppressed the progression and reversed the clinical and histological signs of EAE - The suppressive activity correlated with attenuation of pro-inflammatory Th1 and Th17 cytokines followed by IL-10 induction in MLNs, spleen and blood
Kouchaki et al., 2016 [50]	18 to 55 years, *n* = 30 per group	probiotic capsule: *L*. *acidophilus* *L*. *casei* *B*. *bifidum* *L*. *fermentum* 2 × 10^9^ CFU	12 weeks	B cell- hs-CRP	Decreased serum insulin, hs-CRP- improve B cell function concentration
Salami et al., 2019	20 to 60 years *n* = 24 per group	Probiotic capsules: *B*. *infantis*, *B*. *lactis*, *L*. *reuteri*, *L*. *casei*, *L*. *plantarum* and *L*. *fermentum* 2 × 10^9^ CFU	16 weeks	- IL-6, TNF-α, hs-CRP - IL-10	- Reduction in some of inflammatory markers - Increase in IL-10 and nitric oxide levels

Abbreviations: *CD* cluster of differentiation, *CFU* colony-forming unit, *CNS* central nervous system, *EAE* experimental autoimmune encephalomyelitis, *GATA3* GATA binding protein 3, *GM*-*CSF* granulocyte-macrophage colony-stimulating factor, *hs*-*CRP* high-sensitivity C-reactive protein, *IL* interleukin, *IFN*-*γ* interferon gamma, *MLNs* mesenteric lymph nodes, *MOG* myelin oligodendrocyte glycoprotein, *PLP* proteolipid protein. *ROR*-*γt* RAR-related orphan receptor gamma, *Th* T-helper cells, *TNFα* tumor necrosis factor alpha, *TGF*-*β* transforming growth factor beta; *T*-*reg* regulatory T-cell

### Animal studies

Five animal studies were included in the final evaluation (Table 1). Salehipour et al. [35] investigated the effect of 10^9^ CFU/mL *L*. *plantarum* A7, *B*. *animalis* and a combination of these probiotics in an experimental model of MS. They reported that *L. plantarum* A7 and *B. animals* could ameliorate EAE condition through, ameliorated EAE condition through enhancing the anti-inflammatory cytokines and cell, such as IL (Interleukin)-10, IL-4, and TGF-β; T-regs, CD4+, CD25+, and Foxp3+ in spleen and lymph nodes together with increasing Th2 (T-helper 2) (GATA3); and T-reg (Foxp3) in spleen and brain. Additionally, a decrease in IL-17, IFN-γ (Interferon gamma), IL-6, Th1 (T-bet), and Th17 (ROR-γ) have been observed in all groups. In addition, the lowest level of inflammation and leukocyte infiltration, demyelination, and auto reactive T-cells have been reported by a combination of *L*. *plantarum* A7 and *B*. *animalis*.

Secher et al. [51] indicated that these beneficial traits were associated with a decreased secretion of inflammatory cytokines such as IL-17, IFN-γ, and TNF-α (Tumor Necrosis Factor alpha) and an increase in the production of IL-10 as an anti-inflammatory cytokine by autoreactive CD4+ T-cells, CD4+ Foxp3+ in lymph nodes. Furthermore, these beneficial traits were reportedly associated with decreasing the total numbers of CD4+ and MOG-specific CD4+ T-cells in the SC, in addition to increasing the total numbers of CD4+ and MOG-specific CD4+ T-cells in inguinal, mesenteric, and cervical by 10^8^ CFU/animal *E*. *coli* Nissle 1917. They studied oral administration of the mentioned probiotic strain on infiltration of neuroinflammatory factors and repaired intestinal barrier dysfunction in male mice of 8 to 12 weeks old, which demonstrated that defect in intestinal barrier function could also be treated with *E*. *coli* Nissle 1917 by reducing the FD4 (FITC-Dextran 4 kDa) passage from the intestinal lumen to the blood.

Kobayashi et al. [52] in their work, indicated that 0.6–1.2 × 10^9^ CFU/day/mouse *L*. *casei* strain *Shirota* (LcS) upregulated IL-17 and IFN-γ in ILN cells on days 7 and 12, IL-10, CD4+ CD25+ T-reg cells on day 7, T-reg cells in the spleen on days 7 and 12. Contrary to this, CD8+ T-cells in the spleen on day 12 decreased in LcS group.

Kwon et al. [53] showed that a mixture of five probiotics (IRT5) can ameliorate EAE. Pretreatment and treatment with IRT5 (containing of 10^8^ CFU/g of each strain: *L*. *casei*, *L*. *acidophilus*, *L*. *reuteni*, *B*. *bifidum*, *Streptococcus thermophiles*) suppressed and delayed EAE onset, respectively. Also, authors elucidated that IRT5 powder could enhance IL-4, IL-10 in peripheral CD4+ T-cells and SC, IL-10+ producing CD4+ T-cells in draining lymph nodes (dLN), IL-2 and IL-10 in dLNs (as a site of inflammation) at both mRNA and protein level, and CD4+Foxp3+ T-reg in dLNs. On the other hand, plummeting pro-inflammatory cytokines (Th1/Th17; IFN-γ, TNFα, and IL-17) in peripheral CD4+ T-cells; pathogenic cytokines such as IL-1β, Th1 type (IL-2, IFN-γ, and TNFα) and Th17 type (IL-17) in SC; Th1/Th17 polarization, IL-6, IFN-γ, and TNFα at mRNA level in dLNs; IL-17, IFN-γ, and TNF-α in dLNs at both mRNA and protein level; infiltration of mononuclear cells into the SC, Gr1+, or/and CD11b+ monocyte; and CD4+ T-cells without altering the numbers of B220+ B cells in the SC have been reported in their study.

Similarly, Lavasani et al. [54] exhibited that each monostrain probiotic, individually, failed to perform a therapeutic role in MOG (myelin oligodendrocyte glycoprotein)-induced EAE in female C57BL/6 mice, and also, *L*. *paracasei*, PCC 101 and *L*. *delbrueckii*, DSM 20081 had no effect on the disease development. However, a mixture of the *L*. *paracasei* DSM 13434, *L*. *plantarum* DSM 15312, and *L*. *plantarum* DSM 15313 inhibited the progression of the disease and improved the clinical and histological signs of EAE in mesenteric lymph nodes (MLNs), the spleen, and the blood. Moreover, these probiotics established a variety of effects, such as augmentation of Th2 type cytokines, IL-4, IL-10, TGF-β1, and shifting cytokine profile from Th1 to Th2 in spleen cell cultures with *L*. *paracasei* DSM 1343 and *L*. *plantarum* DSM 15312, IL-10 by MOG-reactive T-cells in spleen cells by lacto-mix against control group, IL-10 producing CD4+T-cells in wild-type (WT) EAE by lacto-mix, IL-27 serum levels in mice fed with *L*. *plantarum* DSM 15313. Despite this, decreasing pro-inflammatory cytokines like IL-17, TNF-α, and IFN-γ from cells and infiltration of CD4+ T-cells in CNS tissues by *L*. *paracasei* DSM 1343 and *L*. *plantarum* DSM 15312, and lacto-mix have been elucidated by this group.

Overall, the animal studies well clarified that supplementation with probiotics could leave a major positive impact on the immune-inflammatory markers, reduce the severity and progression, and also delay the onset of the disease. Based on the animal studies’ reports in this systematic review, it appears that supplementation with probiotics had a modulating effect on the immune activity as well as the inflammatory cytokines.

### Human studies

The two selected human studies were of significant assessment on the effect of probiotics in MS patients (Table 1). Kouchaki et al. [50] investigated the clinical and metabolic response to probiotic supplementation in patients with MS and exhibited that probiotics could alter hs-CRP levels in the probiotic group compared to the placebo group. They demonstrated that B cells’ function was drastically decreased after 12 weeks of receiving a probiotic capsule (*L*. *acidophilus*, *L*. *casei*, *B*. *bifidum* and *L*. *fermentum*).

In another study, Salami et al. assessed inflammatory biomarkers before and after 16 weeks of supplementation with a probiotic capsule comprising *B*. *infantis*, *B*. *lactis*, *L*. *reuteri*, *L*. *casei*, *L*. *plantarum*, and *L*. *fermentum* (2 × 10^9^ CFU/day). Furthermore, they fed the control group with capsules containing maltodextrine, and the final size of each group was 24 persons per group. They were able to prove a crucial increase of serum IL-10 level. Contrary to this, the probiotic supplement remarkably decreased IL-6 and hs-CRP levels. However, no meaningful alteration in TNF-α was found after adjustment for age and BMI.

Contrary to the animal studies, the number of human studies were limited; however, according to the evidence of these two studies, it could be concluded that supplementation with probiotics may have beneficial effects on the immune function and especially inflammatory parameters. Nevertheless, more RCTs articles were needed to reach a rather satisfactory conclusion.

## Discussion

In the present systematic review, the effects of several probiotic supplementations on immune and inflammatory responses in animal and human models of MS were surveyed. The majority of the reviewed studies supported the beneficial effects, including alleviation, prevention, and delaying the onset of diseases via improving immune and inflammatory mechanisms (Table 1). Therefore, it seems that immunological and biological effects of probiotics through increasing anti-inflammatory cytokines and T-reg cells, along with reducing pro-inflammatory cytokines, are the prominent mechanisms that have been demonstrated. An increase in the incidence and mortality rates related to MS remains high in the world. On the other hand, taking probiotic supplements for health and other purposes is growing. In this review, it was observed that consumption of probiotics has beneficial effects on systemic and central inflammation, as well as immune control in EAE models and MS patients.

Analysis of the method and result sections of the reviewed articles revealed that 10^9^ CFU/mL dosage was more effective than other dosages. It is undeniably vital to note that the type of consumed probiotics species are of major significance, and the type of strains and host specificity are the most eminent factors influencing probiotics efficacy [55, 56]. Difference in environmental conditions and diet could substantially influence the results of studies. Therefore, it was not feasible to pinpoint which species could be more effective. However, in two studies, the effects of several different species on the immune and inflammatory parameters were evaluated [35, 50, 53, 54]. It was suggested that the combination of several probiotics has a stronger effect than their separate use [57, 58].

Another critical point is the duration of the interventions in human and animal reports, which is reported from 3 to 16 weeks, independent of the dosage and type of species. This systematic review indicates that the 8–12-week results were more favorable. Stronger results were reported within this period. In some cases, improvement of physiological responses was observed in 2 or 3 weeks, and favorable results were reported in relation to immunity and inflammation [59, 60].

**Fig. 2:**
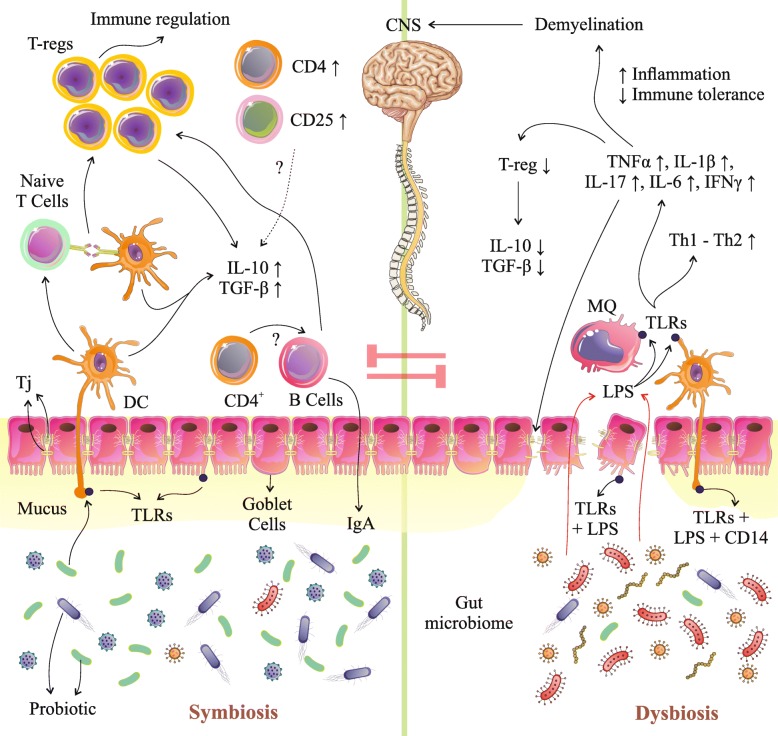
Probiotic mechanisms in the gastrointestinal tract. Probiotics administration may manipulate gut microbial composition and reduce growth of pathogens and stimulate mucin secretion via stimulation of Goblet cells. Probiotic-mediated immunomodulation may occur through mediation of cytokine secretion through TLRs (ECs, DCs, and MQs), which can also affect proliferation and differentiation of immune cells (T-cells, CD cells, B cells) especially T-reg cells which resulted in an increase of IL-10 and TGF-β. Also, probiotics, through amplification of TJs, could improve gut permeability and also decrease entrance of LPS and pathogens from lumen into circulation. T-reg, regulatory T-cell; TLRs, toll-like receptors; ECs, endothelial cells; DCs, dendritic cells; MQs, macrophage cells; IL, interleukin; CD, cluster of differentiation; TGF-β, transforming growth factor beta; TNF-α, Tumor necrosis factor alpha; IFN-γ, Interferon gamma; TJPs, tight junction proteins; Ig, immunoglobulin g

Findings from observational studies and published RCTs suggest that several possible factors are responsible for attenuation of the association between probiotics administration and observed MS features. It was a proposed mechanism related to the effects of probiotics administration and immune and inflammatory response based on the included studies in this review (Fig. 2), but further investigations are still required.

### Proposed mechanisms of probiotics in modulating immune and inflammatory responses

Taken together, animal and human studies present that probiotics may play an acute role in the modulation of immune and inflammatory mechanisms in MS; however, the clear mechanisms have not been well defined yet. The effects of probiotics intake could be confounded by diet [61], age [62], BMI (body mass index) [63, 64], drugs [65], and stress [66]. All of these agents hold dire implications for the gut microbiome composition in addition to host gut functions (permeability and physiology). Evidence has demonstrated that the use of probiotics could improve the gut microbial population [21, 67], increasing mucus-secretion [68], and also prevent the destruction of tight junction proteins (TJs) [69] via a decrease in the amount of lipopolysaccharides (LPS). When LPS binds to toll-like receptors (TLR 2, 4) on endothelial cells (ECs), DCs (dendritic cells) and macrophage cells (MQs) are activated and inflammatory markers increase [21, 44, 70, 71]. Finally, a reduction of gut dysbiosis and gut leaky after probiotic therapy could decrease the production of inflammatory biomarkers and blunt excessive immune system stimulation (Fig. 2) [21, 27, 72–74]. Based on the studies cited in this review, differentiation of T-cells toward Th2 and also the production of Th2 cytokines like IL-10 and IL-4 are augmented by probiotics [53, 54]. Furthermore, another mechanism that can participate in the progression of EAE is associated with the expression of transcription factors for Th1 and Th17. Hence, the administration of probiotics would downregulate these factors [53]. Furthermore, oral supplementation with probiotics in the EAE model not only provoked the production of TGF-β and IL-10, but also increased the number of T-reg cells [44]. In particular, this reaction is simultaneous with the increasing of Foxp3 [35, 52–54]. Ultimately, it could be demonstrated that an upward trend in the expression of Foxp3 and GATA3 vs. a downward trend in expression of T-bet and ROR-γt are the primitive molecular immunosuppression mechanisms, which regulate the equilibrium between Th1/Th2 and Th17/T-reg toward a Th2 and T-reg response. However, the authors mentioned that they would evaluate the role of DCs and epigenetic changes in both therapeutic and prophylactic models [35, 54]. To be more precise, the secretion of IFN-γ, IL-17, GM-CSF, and TNFα was decreased, while the production of IL-10 due to an enriched population of CD4+CD25+Foxp3+T-regs was increased. Also, it was verified that oral administration of probiotics could reduce MOG-reactive T-cell proliferation and pro-inflammatory cytokine levels and increase IL-10 + or/and Foxp3+T-reg cells [54].

### Limitations

Studies included in this systematic review had several limitations. (1) The gut microbiome composition has multiple differences and variations among people and animals, which may be due to differences in diet, environmental and personal characteristics, and even genetic factors, and gut microbiome, in these studies, was not evaluated directly [29]. (2) The information on the gut microbiome population is still unclear in diseases [75]. In spite of the remarkable advances in tools and technology in bioinformatics, there are still some limitations in this field. In connection with the identification of probiotics intake on gut-related mechanisms for their effects, more extensive research is needed [75]. 3) The effects of the diet on the efficiency of probiotics administration as well as gut microbiome composition are very strong [76, 77]. Therefore, in human studies, attention to the dietary differences of participants is highly essential for observing the net outcome of interventions [78]. (4) The use of various drugs, especially antibiotics drugs, can directly or indirectly affect the gut microbiome, whose effects in human studies are not well controlled [79]. Altogether, there were consistent results in human and animal studies, but the findings of human studies were likely to be less accurate than animal studies. Some possible reasons are listed below. Differences in the type of the parameters in different tissues like brain, SC, blood, etc.; the various supplemental dosages; type of species; and samples (gender and age) are due to disrupt the clear and accurate comparison of the findings among studies. In addition, human studies were very limited in this field.

## Conclusion

Few investigations have specifically assessed the effect of probiotic supplements on MS in animals and humans. To date, interventional studies’ evidences suggest that probiotic supplementation may have beneficial effects on reducing the risk and preventing and delaying MS. This paper suggests that administration of probiotics positively affects CNS disorder and demyelination process, especially via gut microbiome composition improvements, which is yet to be investigated. Future studies of probiotic supplementation using particularly large-scale RCTs with adequate dosages and sample sizes in MS are required to elucidate the potential role of probiotic therapy on promotion of the immune system and suppressing inflammation.

## Supplementary information

**Additional file 1.** Quality assessment of randomized controlled trials and animal studies.

## Data Availability

All data generated or analyzed during this study are included in this published article.

## Abbreviations

CD: Cluster of differentiation
CFU: Colony-forming unit
CNS: Central nervous system
EAE: Experimental autoimmune encephalomyelitis;
EDSS: Expanded disability status scale
GATA3: GATA binding protein 3
GM-CSF: Granulocyte-macrophage colony-stimulating factor
hs-CRP: High-sensitivity C-reactive protein
IFN-γ: Interferon gamma
IL: Interleukin
LPS: Lipopolysaccharide
MLNs: Mesenteric lymph nodes
MOG: Myelin oligodendrocyte glycoprotein; Multiple Sclerosis (MS)
PLP: Proteolipid protein
PRISMA: Preferred Reporting Items for Systematic Reviews and Meta-Analyses
ROR-γt: RAR-related orphan receptor gamma
SCFAs: Short-chain fatty acids
TGF-β: Transforming growth factor beta
Th: T-helper cells
TLRs: Toll-like receptors
TNFα: Tumor necrosis factor alpha
T-reg: Regulatory T-cell
