# Supplementary material for: Immunomodulatory and anti-inflammatory effects of probiotics in multiple sclerosis: a systematic review
Source: J Neuroinflammation. 2019 Nov 21;16:231. doi: 10.1186/s12974-019-1611-4 (PMC6868771; doi:10.1186/s12974-019-1611-4)
Supplement: Supplementary file 1 — Additional file 1. Quality assessment of randomized controlled trials and animal studies. [file 12974_2019_1611_MOESM1_ESM.pdf]

Cochrane Risk of Bias Tool to AHRQ Standards (Good, Fair, and Poor)

Author, Year	Randomization adequate?	Allocation concealment adequate?	Groups similar at baseline?	Outcome assessors blinded?	Clinician blinded?
Salami, 2019	✓	✓	✓	✓	✓
Kuchaki,2016	✓	✓	✓	✓	✓

Author, Year	Patient or caregiver blinded?	Intention to treat?	Acceptable level of *Overall* attrition (≤20%)?	Acceptable level of *differential* attrition (<10%)?	Overall quality (Good, Fair, Poor)
Salami, 2019	✓	✓	✓	✓	Good
Kuchaki,2016	✓	✓	✓	✓	Good

SYRCLE`s tool for assessing risk of bias for animal studies

Item	Type of bias	Domain	Description of domain	Review authors judgment	Studies
1	Selection bias	Sequence generation	Describe the methods used, if any, to generate the allocation sequence in sufficient detail to allow an assessment whether it should produce comparable groups.	Was the allocation sequence adequately generated and applied? (*)	7 (×)<br>6 (×)<br>5 (×)<br>2 (×)<br>3 (*)
2	Selection bias	Baseline characteristics	Describe all the possible prognostic factors or animal characteristics, if any, that are compared in order to judge whether or not intervention and control groups were similar at the start of the experiment.	Were the groups similar at baseline or were they adjusted for confounders in the analysis?	7 (×)<br>6 (×)<br>5 (×)<br>2 (×)<br>3 (*)
3	Selection bias	Allocation concealment	Describe the method used to conceal the allocation sequence in sufficient detail to determine whether intervention allocations could have been foreseen before or during enrolment.	Was the allocation adequately concealed? (*)	7 (*)<br>6 (×)<br>5 (×)<br>2 (×)<br>3 (*)
4	Performance bias	Random housing	Describe all measures used, if any, to house the animals randomly within the animal room.	Were the animals randomly housed during the experiment?	7 (×)<br>6 (×)<br>5 (×)<br>2 (×)<br>3 (*)

Item	Type of bias	Domain	Description of domain	Review authors judgment	Studies
5	Performance bias	Blinding	Describe all measures used, if any, to blind trial caregivers and researchers from knowing which intervention each animal received. Provide any information relating to whether the intended blinding was effective.	Were the caregivers and/or investigators blinded from knowledge which intervention each animal received during the experiment?	7 (*)<br>6 (×)<br>5 (×)<br>2 (×)<br>3 (*)
6	Detection bias	Random outcome assessment	Describe whether or not animals were selected at random for outcome assessment, and which methods to select the animals, if any, were used.	Were animals selected at random for outcome assessment?	7 (×)<br>6 (×)<br>5 (×)<br>2 (×)<br>3 (×)
7	Detection bias	Blinding	Describe all measures used, if any, to blind outcome assessors from knowing which intervention each animal received. Provide any information relating to whether the intended blinding was effective.	Was the outcome assessor blinded?	7 (*)<br>6 (×)<br>5 (×)<br>2 (×)<br>3 (*)
8	Attrition bias	Incomplete outcome data	Describe the completeness of outcome data for each main outcome, including attrition and exclusions from the analysis. State whether attrition and exclusions were reported, the numbers in each intervention group (compared with total randomized animals), reasons for attrition or exclusions, and any re-inclusions in analyses for the review.	Were incomplete outcome data adequately addressed? (*)	7 (*)<br>6 (*)<br>5 (*)<br>2 (*)<br>3 (*)

Item	Type of bias	Domain	Description of domain	Review authors judgment	Studies
9	Reporting bias	Selective outcome reporting	State how selective outcome reporting was examined and what was found.	Are reports of the study free of selective outcome reporting? (*)	7 (*)<br>6 (*)<br>5 (*)<br>2 (*)<br>3 (*)
10	Other	Other sources of bias	State any important concerns about bias not covered by other domains in the tool.	Was the study apparently free of other problems that could result in high risk of bias? (*)	7 (×)<br>6 (×)<br>5 (×)<br>2 (×)<br>3 (*)

* Is point 1

× Is point 0
